# Development and characterization of a thermo-responsive, CT-detectable, biocompatible fiducial marker for potential application in image-guided radiotherapy

**DOI:** 10.1016/j.bbiosy.2026.100153

**Published:** 2026-10-26

**Authors:** Saeideh Arsalani, Alireza Zomorodian, Mina Maksimos, Saad Asim, Tithi Bhattacharyya, Yun-Cheng Liu, Obed Asare, Feda Azab, Anne M. Alsup, Muhammad Rizwan, Emina Huang, Abdel Kareem Azab

**Affiliations:** a Department of Biomedical Engineering, University of Texas Southwestern Medical Center, Dallas, TX, USA; b Department of Internal Medicine, University of Texas Southwestern Medical Center, Dallas, TX, USA; c Charles and Jane Pak Center for Mineral Metabolism and Clinical Research, University of Texas Southwestern Medical Center, Dallas, TX, USA; d Harold C. Simmons Comprehensive Cancer Center, University of Texas Southwestern Medical Center, Dallas, TX, USA; e Department of Surgery, University of Texas Southwestern Medical Center, Dallas, TX, USA

**Keywords:** PNIPAM, Barium sulfate, CT marker, Tumor localization, Colorectal cancer, Image-guided radiation therapy

## Abstract

Radiotherapy remains underutilized in colon cancer despite the intrinsic radiosensitivity of colon cancer cells, largely because accurate targeting of tumors within the highly mobile colon is technically challenging. Tumor motion caused by peristalsis, bowel motility, and respiration limits the precision of radiation delivery and increases the risk of damage to surrounding radiosensitive tissues. Current endoscopic tattooing agents used for tumor localization are used for operative localization only and lack radiographic visibility, may diffuse from the injection site, and can induce local inflammation.

Here, we developed a thermo-responsive, CT-detectable, injectable fiducial marker composed of Poly(N-isopropylacrylamide) (PNIPAM) loaded with barium sulfate (BaSO₄) for potential application in tumor localization during image-guided radiotherapy. The PNIPAM-BaSO₄ formulation remained injectable at room temperature and rapidly underwent in situ gelation within approximately 15 s at physiological temperature. Micro-CT imaging demonstrated strong dose-dependent radiopacity both in vitro and in vivo, supporting the potential of the marker for CT-based visualization. The marker exhibited stable mass retention for up to 280 days in vitro, and maintained stable localization and radiographic signal for 3 months in vivo without detectable diffusion. Histopathological analysis revealed no evidence of local inflammation, fibrosis, necrosis, or systemic toxicity, demonstrating excellent biocompatibility.

Collectively, these findings establish the feasibility of the PNIPAM-BaSO₄ platform as a stable, CT-detectable fiducial marker and support its further development for potential application in image-guided radiotherapy. Future studies will evaluate its performance under clinically relevant radiotherapy imaging and treatment conditions, including its potential use for interfractional localization of mobile tumors.

## Highlights

• Developed a thermo-responsive CT-visible injectable fiducial marker.
• PNIPAM-BaSO₄ marker rapidly solidifies in situ at body temperature.
• Marker demonstrated long-term stability and strong CT radiopacity.
• PNIPAM-BaSO₄ showed excellent local and systemic biocompatibility.
• Platform may enable radiotherapy for highly mobile tumors such as colon cancer.

## Graphical abstract

**Figure:**
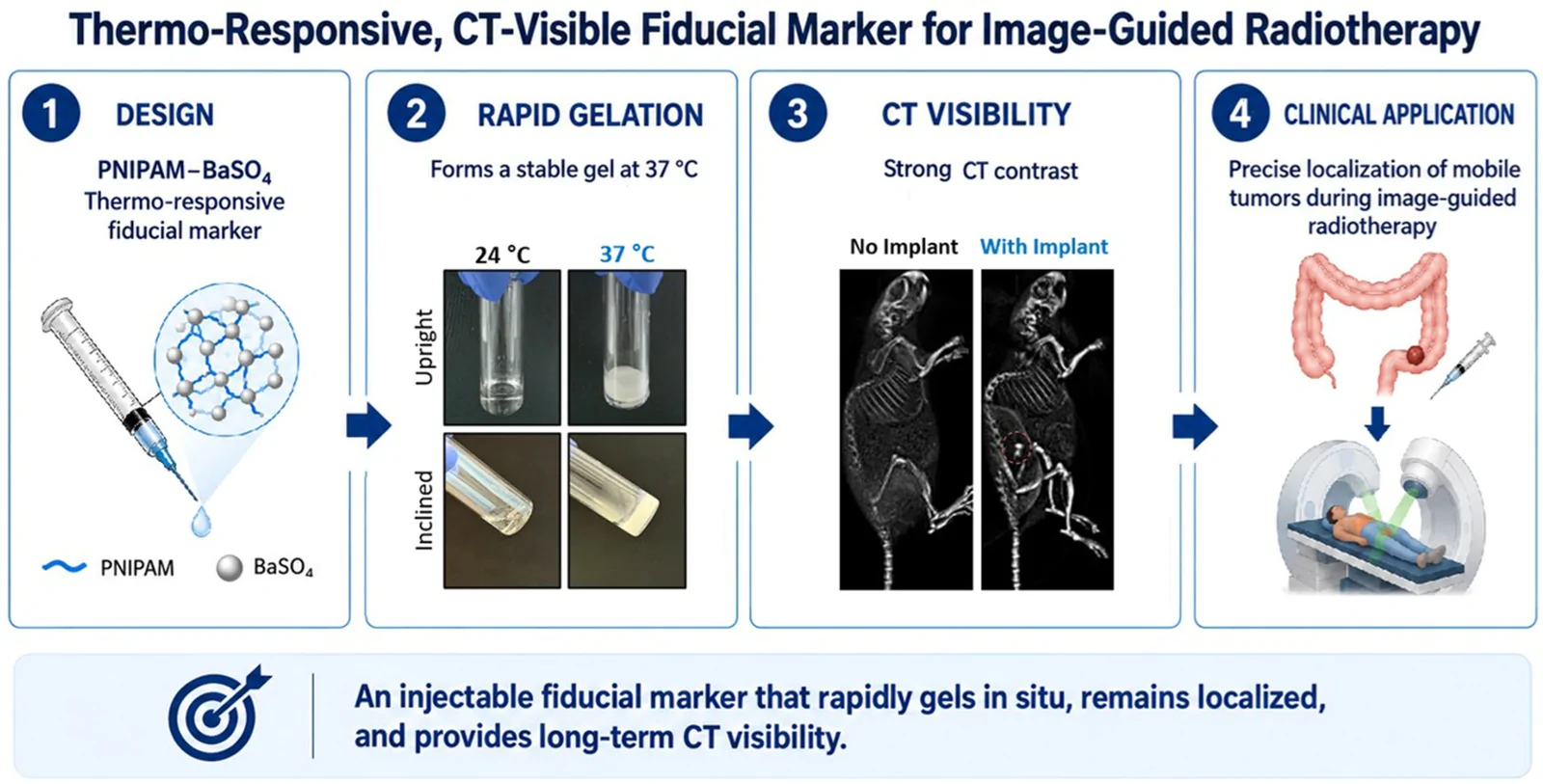

## 1 Introduction

The gastrointestinal (GI) tract cancers account for approximately 24% of global cancer incidence and 33% of cancer-related deaths [1]. According to the World Health Organization (WHO) and Global Cancer Observatory (GLOBOCAN 2024), colorectal cancer (CRC) is the third most diagnosed cancer and the second leading cause of cancer mortality, accounting for over 1.9 million new cases and 930,000 deaths annually [2] with incidence rates projected to increase by 60% for colon and 72% for rectal by 2035 [3].

Using the TNM staging system, colon cancer progresses in a predictable clinical continuum [[4], [5], [6]]. Stage 0 and Stage I (no lymph node involvement) are managed with surgical resection alone, yielding an excellent prognosis with over 90–95% 5-year survival. Stage II (no lymph node involvement) and Stage III (involvement of local and regional lymph nodes) are treated with surgery combined with adjuvant chemotherapy, leading to generally good but variable 75% and 50% survival, respectively. Stage IV represents metastatic disease to distant organs and is treated with systemic therapy (chemotherapy, targeted agents, and/or immunotherapy) with a significantly lower prognosis. In addition to surgery (a loco-regional intervention), loco-regional stages of cancer, such as breast, lung, cervix, prostate, and head and neck cancers [[7], [8], [9], [10], [11]], and even rectal cancer [12,13], are treated mainly with other loco-regional interventions (radiotherapy, with or without chemotherapy), while loco-regional stages II-III of colon cancer are treated mainly with systemic interventions (chemotherapy).

Radiation therapy is rarely used for colon cancer [14,15], despite accumulating evidence in the literature that colon cancer cells, similar to rectal cancer, are highly sensitive to radiation in vitro [[16], [17], [18]]. The primary limitation of the use of radiotherapy for colon cancer is the high mobility of the colon caused by peristalsis, motility, and breathing [19,20], along with the high sensitivity of adjacent normal tissues to radiation [21]. Thus, there remains an unmet clinical need for strategies that improve tumor localization to allow precise targeting of radiotherapy to colon cancer, at loco-regional stages (Stage II-III) of the disease.

For tumor localization of external tumors, such as breast, head and neck cancers, and skin cancers, skin-based markers (e.g., tattoos) are commonly used to guide radiation therapy by ensuring consistent patient positioning across treatment sessions [22,23]. However, such markers cannot be used to determine the location of internal tumors. For internal organs such as the colon, conventional endoscopic tattooing using carbon-based dyes (e.g., India ink, methylene blue, and indocyanine green) are widely used to mark tumor sites before surgery. This approach helps surgeons identify lesions, avoid resection of incorrect regions, and improve surgical efficiency [24,25]. However, these inks are not visible using clinically available imaging modalities, they diffuse around tissues, they fade over a short period of time, and in some cases, they may cause inflammation and peritonitis [26,27].

Therefore, there is an urgent unmet clinical need for a GI fiducial marker that is visible across clinically available imaging modalities, remains stably localized within tissue, and is biocompatible, thereby improving the accuracy of tumor localization and enabling effective radiation delivery. In this study, we hypothesized that Poly(N-isopropylacrylamide) (PNIPAM)-based implant loaded with barium sulfate (BaSO_4_ ) will provide a thermo-responsive, CT-detectable, biocompatible fiducial marker for precise positioning of mobile tumors, such as colon cancer, in image-guided radiotherapy.

## 2 Methods

### 2.1 Materials

Poly(N-isopropylacrylamide) PNIPAM (Cat. # 535,311), barium sulfate (BaSO_4_) (Cat. # 243,353), and glycerol (Cat. # G7893), were purchased from Millipore-Sigma (St. Louis, MO). Seven weeks C57BL/6 mice (Stock No: 000,664) were purchased from Jackson Laboratories (Bar Harbor, ME). All animal experiments were performed in accordance with protocols approved by the Institutional Animal Care and Use Committee (IACUC) at UT Southwestern Medical Center (protocol # 103,868) and complied with all relevant institutional and national guidelines for the care and use of laboratory animals.

### 2.2 Preparation of the PNIPAM-BaSO_4_ marker

PNIPAM was used as the thermo-responsive polymer, and BaSO₄ was incorporated as a radiopaque contrast agent. PNIPAM was dissolved in phosphate-buffered saline (PBS) to serve as a thermo-responsive vehicle. BaSO₄ is not soluble in water [21], therefore, it was first wetted with glycerol, then suspended in PBS (PBS: glycerol 1:1 final ratio). Barium sulfate stock was gradually incorporated into the PNIPAM stock at a final ratio of 1:10 by gentle mixing over a medium-speed vortex to give the final PNIPAM-BaSO_4_ formulation.

### 2.3 Rheological characterization of injectability

Rheological testing was conducted on PNIPAM solutions at concentrations ranging from 100–250 mg/ml at room temperature (24 °C) using a rheometer (MCR 302, Anton Paar, Austria). Also, a practical demonstration of manual injection was performed and photographed, where a 150 mg/ml PNIPAM/PBS formulation was injected through a 25 G syringe at room temperature.

### 2.4 Solidification kinetics and volume

A room-temperature solution of 150 mg/ml PNIPAM (1000 μl) in a glass test tube was placed in a physiological body temperature (37 °C) water bath, and the time of complete solidification of the solution was recorded to quantify the thermo-responsiveness of the solution. In addition, a qualitative demonstration of the thermo-responsive behavior of the PNIPAM solution was performed and photographed.

To assess the relationship between the liquid PNIPAM volume and the size of the thermally formed gel, 10, 25, and 50 µL of PNIPAM solution were dispensed onto a flat surface placed on a hot plate pre-equilibrated to 37 °C. Upon exposure to 37 °C, the thermo-responsive PNIPAM rapidly underwent gelation, and images of the resulting gels were acquired. Gel dimensions were determined, and gel volume (half sphere) was calculated.

### 2.5 CT imaging

#### 2.5.1 In vitro

To establish CT visibility in vitro, PNIPAM-BaSO_4_ marker with increasing (0, 0.1, 0.5, 1, 5, and 10 mg/ml) BaSO₄ concentrations (in 150 mg PNIPAM/ml PBS) were prepared. For each concentration, 150 mg/ml of the preparation was transferred into each well of a 96-well plate, and micro-CT imaging was then performed using an IVIS Spectrum CT (PerkinElmer/Revvity, Waltham, MA, USA), and the mean Hounsfield Unit (HU) values were measured to quantify the relationship between BaSO₄ concentration and radiographic intensity.

#### 2.5.2 Ex vivo

For CT visibility of PNIPAM-BaSO₄ marker ex vivo, 5 g of ground meat was placed into each well of a 6-well plate and incubated at 37 °C for 2 h. Increasing volumes (0–100 µL) of the PNIPAM-BaSO₄ marker were then injected into the center of the meat. Temperature was maintained at 37 °C, and micro-CT imaging was performed 1 h post-injection, and the mean HU values were measured to assess radiopacity, localization, and visibility in a soft tissue-like environment.

#### 2.5.3 In vivo

For CT visibility of the PNIPAM-BaSO_4_ marker in vivo, increasing volumes (0–50 µl) of the PNIPAM-BaSO_4_ marker were injected subcutaneously in C57BL/6 mice. Micro-CT imaging was performed 1 h post-injection, and the mean HU values were measured to assess radiopacity, localization, and visibility in vivo.

### 2.6 Stability

#### 2.6.1 In vitro

For in vitro stability, 100 µl of the PNIPAM-BaSO_4_ marker was incubated in test tubes at 37 °C for 1 min to induce gelation, and then 1 mL of PBS (37 °C) was added and incubated for 0, 1, 3, 7, 14, 30, 60, 90, 120, 180, and 280 days. At each time point, PBS was removed, the implant was paper-dried and weighed, and the results were normalized to time zero.

#### 2.6.2 In vivo

For in vivo stability, 50 µL of the PNIPAM-BaSO_4_ marker was injected subcutaneously into C57BL/6 mice. Micro-CT imaging was performed 1 day, 4 weeks, and three months after injection, the mean HU values were measured, and these were normalized to the values of Day 1.

### 2.7 Biocompatibility in vivo - histopathological analysis

For biocompatibility in vivo, 50 µL of the PNIPAM-BaSO_4_ marker was injected subcutaneously into C57BL/6 mice and left for 3 months. Then, mice were sacrificed, and their organs (skin and muscle from the implantation site, skin and muscle from the opposite side with no implant, heart, kidney, liver, lung, and spleen) were harvested. Organs were then rinsed in PBS and fixed in 10% neutral-buffered formalin. Fixed tissues were then processed, embedded in paraffin, and sectioned into 4–5 μm slices. Sections were mounted on glass slides, deparaffinized, and subjected to hematoxylin and eosin (H&E) staining, followed by histological evaluation. Quantification of histological scores of inflammation, fibrosis/fibroplasia, necrosis/ injury, edema/hemorrhage, and architectural disruption in the skin and muscle tissues adjacent to the implantation site or control PBS injection under the skin was performed on a scale of 0 to 4 (0= absent; 1=minimal, 2=mild, 3=moderate, and 4=severe). Scores were presented as the average for each group.

### 2.8 Cytotoxicity assay of PNIPAM-BaSO4 on SW480 & COLO 205

SW480 and COLO 205 human colorectal cancer cell lines were used to evaluate the cytotoxic effects of the PNIPAM-BaSO₄ formulation. Cells were seeded in 96-well plates at a density of 50 K cells/well and allowed to adhere overnight. Different volumes of the PNIPAM-BaSO₄ formulation (0, 2.5, 5, 10, and 25 μL) were prepared and incubated overnight at 37 °C to allow gelation. The resulting gels were then transferred to the corresponding wells, and incubated for 24 h. Cell viability was subsequently assessed using an MTT assay. Briefly, 10 μL of MTT solution (0.5 mg/mL) was added to each well and incubated for 4 h to allow the formation of formazan crystals. The resulting crystals were dissolved in a 1:1 (v/v) mixture of DMSO and methanol, and absorbance was measured at 570 nm using a microplate reader.

### 2.9 Statistical analysis

Data are presented as mean ± standard deviation (SD). Statistical analyses were performed using GraphPad Prism. Group differences were analyzed using one-way analysis of variance (ANOVA), and individual points were compared using student's t-test. Dose-response relationships were evaluated by linear regression with the coefficient of determination (R²), and significance was reported. Each group included at least three independent biological replicates, specific numbers of replicates for each experiment were added in figure legends. A p-value <0.05 was considered statistically significant.

## 3 Results

### 3.1 PNIPAM-BaSO_4_ marker showed injectable properties at room temperature

We tested the viscosity of PNIPAM solution as a function of shear rate, at room temperature. The green-shaded area shows the injectable range, while the red-shaded area shows the non-injectable range, with a threshold of 120 mPa.s [28]. PNIPAM solutions 100 and 150 mg/ml exhibited injectable viscosities, whereas higher concentrations (200–250 mg/ml) demonstrated non-injectable viscosity. Therefore, we used the highest injectable concentration possible (150 mg/ml) (Fig. 1**Ai**). A visual demonstration of the smooth injection of 150 mg/ml PNIPAM formulation through a syringe (Fig. 1**Aii**).

**Fig. 1:**
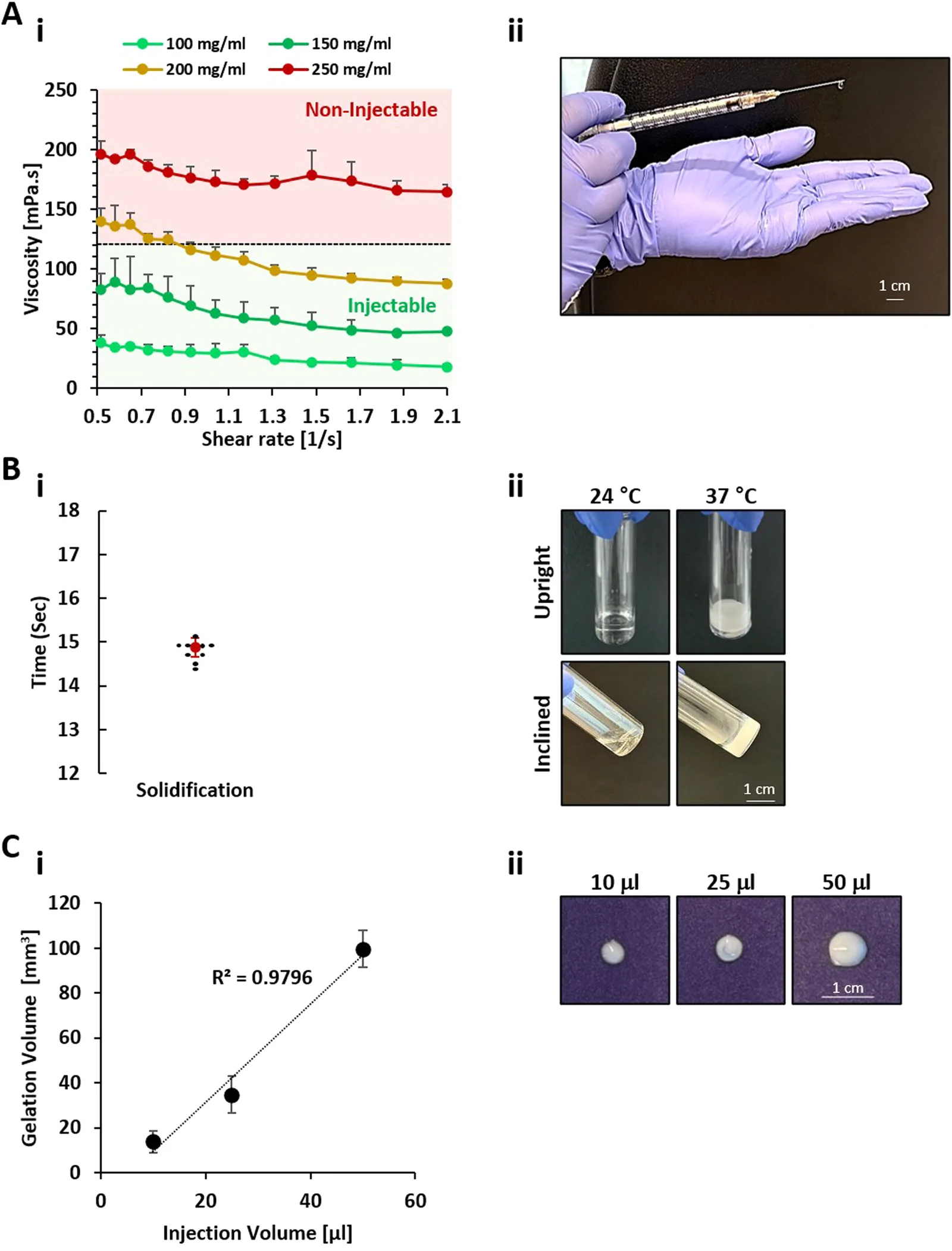
The injectability, gelation time, and thermo-responsive behavior of the PNIPAM-BaSO_4_ marker.

Viscosity behavior of PNIPAM at different concentrations as a function of shear rate, the dashed black line indicates the injectability threshold (around 120 mPa.s) (Ai) [Each data point represents the average ± SD of 3 independent replicates]. The image depicts the injectability of the PNIPAM polymer formulation using a syringe (Aii). Gelation time of 150 mg/ml of PNIPAM (Bi) [Each data point represents the average ± SD of 10 independent replicates]. Transition of PNIPAM in upright and inclined tubes from liquid at 24 °C to solid phases at 37 °C (Bii). Effect of initial PNIPAM volume on the resulting hydrogel volume following thermal conversion (Ci) [Each data point represents the average ± SD of 5 independent replicates]. Representative photographs of the hydrogels formed from 10, 25, and 50 µL of liquid PNIPAM, illustrating the corresponding increase in gel size (Scale bar = 1 cm) (Cii).

### 3.2 PNIPAM-BaSO_4_ marker solidified quickly at body temperature (37 °C)

The solidification kinetics of 150 mg/ml PNIPAM at 37 °C were determined. A rapid phase transition from liquid to solid-gel, was observed at approximately 15 s after exposure to a 37 °C water bath (Fig. 1**Bi**). A visual demonstration of the thermos-responsive behavior of the PNIPAM solution in both upright and inclined tubes showed a free-flowing liquid at 24 °C, in contrast to a non-flowing solid-gel at 37 °C (Fig. 1**Bii**), enabling in situ solidification. In addition, a video demonstrating the transition from liquid (at room temperature) to solid/gel (in a 37 °C water bath) was presented (**Supplementary Fig. 1).**

Increasing the liquid PNIPAM volume (10, 25 and 50 µL) resulted in a corresponding increase in the volume of the resulting hydrogel after thermal conversion, with a strong linear correlation (R²=0.9796), (Fig. 1**Ci**). A visual demonstration of the different PNIPAM hydrogel volumes formed was shown in (Fig. 1**Cii**). These findings indicate that the final gel volume increased proportionally with the amount of PNIPAM initially applied.

### 3.3 PNIPAM-BaSO_4_ marker showed dose-dependent CT signal, in vitro, ex vivo and in vivo

In vitro, a strong positive correlation was observed between BaSO₄ concentration suspended in a 150 mg/ml PNIPAM and micro-CT signal (R² = 0.97, p = 3.1 × 10⁻⁴), indicating a significant dose-dependent opacity (Fig. 2**Ai**), and representative images of the micro-CT imaging are shown in Fig. 2**Aii**. Therefore, for the subsequent experiments, the highest concentration of BaSO₄ of 10 mg/ml was utilized. Ex vivo, a strong positive correlation was observed between injected volume and micro-CT signal (R² = 0.97, p = 4.4 × 10⁻⁴), indicating a significant dose-dependent radiopacity (Fig. 2**Bi**), and representative images of the micro-CT imaging are shown in Fig. 2**Bii**. In vivo, a strong positive correlation was observed between injected volume and micro-CT signal (R² = 0.95, p = 0.025), indicating a significant dose-dependent opacity (Fig. 2**Ci**), and representative images of the micro-CT imaging are shown in Fig. 2**Cii**.

**Fig. 2:**
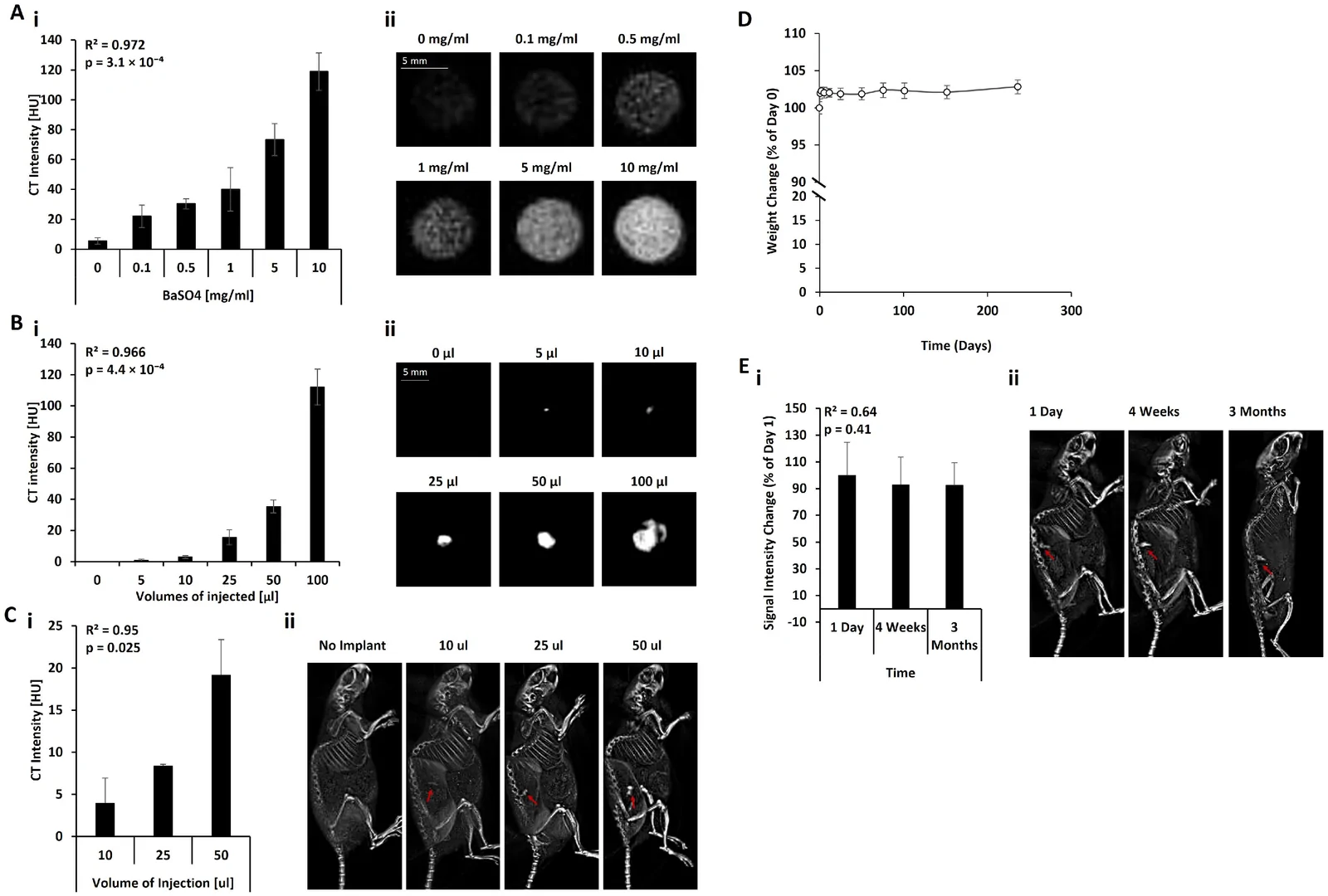
CT visibility and stability of the PNIPAM-BaSO₄ marker.

Micro-CT signal increases with BaSO₄ concentration (R² = 0.972, p = 3.1 × 10⁻⁴) (Ai). Representative micro-CT images of different BaSO₄ concentrations (Aii) [Each data point represents the average ± SD of 3 independent replicates]. Ex vivo correlation between implant volume and CT signal (R² = 0.966, p = 4.4 × 10⁻⁴) (Bi) [Each data point represents the average ± SD of 3 independent replicates]. Representative micro-CT images of meat injected with increasing volumes (Bii) [Each data point represents the average ± SD of 3 independent replicates]. In vivo correlation between implant volume and CT signal (R² = 0.95, p = 0.025) (Ci). Representative micro-CT images of subcutaneous implants in mice (Cii). In vitro mass retention of the implant was over 280 days. No correlation between time and mass was observed (R^2^=0.1, p = 0.3) (D) [Each data point represents the average ± SD of 6 independent replicates]. In vivo CT signal stability over 3 months (Ei) [Each data point represents the average ± SD of 3 independent replicates]. Representative micro-CT images at 1 day, 4 weeks, and 3 months, with red arrows marking the implant location. No significant changes in the micro-CT signal were observed over time (R² = 0.64, p = 0.41) (Eii).

### 3.4 PNIPAM-BaSO_4_ marker showed long-term stability, in vitro and in vivo

In vitro, an initial increase in weight (indicating hydration) was observed between Day 0 and Day 1 (p < 0.001), followed by stable signal levels with no significant changes from Day 1 through Day 280 (p = 0.3), (Fig. 2**D**), no detectable degradation was observed under the conditions tested. In vivo, the micro-CT signal remained stable over time, with no significant differences between 1 day, 4 weeks, and 3 months (p = 0.41), indicating long-term stability (Fig. 2**Ei**), relative stability was observed under the conditions tested. Representative images of the micro-CT imaging are shown in Fig. 2**Eii**.

### 3.5 PNIPAM-BaSO_4_ marker showed biocompatibility, in vitro and in vivo

(Fig. 3**A**) Low-magnification (5 ×) and (Fig. 3**B**) higher-magnification (22 ×) H&E-stained sections of naïve skin and skin from the area injected with PNIPAM-BaSO_4_ marker. At both magnifications, tissues exhibit comparable histological architecture, including intact epidermal and dermal layers with well-preserved underlying structures. No evidence of inflammatory cell infiltration, fibrosis, necrosis, or structural disruption was observed, indicating no detectable histopathological differences between groups. No detectable toxicity was observed under the conditions tested.

**Fig. 3:**
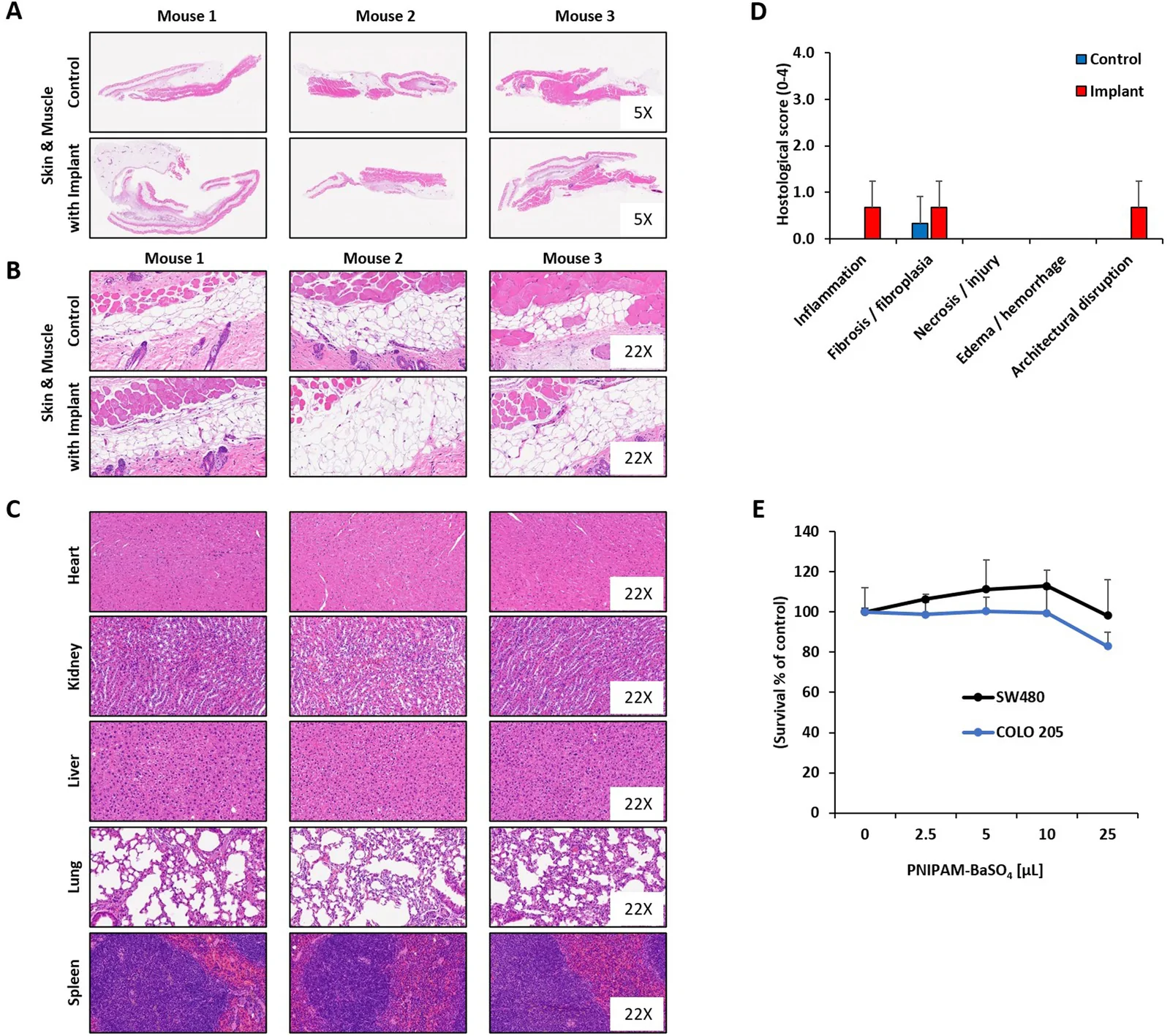
Histological and cytotoxicity assessment of the PNIPAM-BaSO₄ marker.

H&E images show no foreign body reaction or inflammation at the implantation site of the PNIPAM-BaSO₄ marker compared to controls at 5 × (A) and 22x (B). Distal organs (heart, kidney, liver, lung, spleen) exhibit normal morphology with no systemic toxicity (22 ×) (C). Quantification of histological scores of inflammation, fibrosis/fibroplasia, necrosis/ injury, edema/hemorrhage, and architectural disruption in tissues adjacent to the implantation site or control PBS injection under the skin (D) [Each data point represents the average ± SD of 3 independent replicates]. MTT cell viability assay of SW480 and COLO 205 cells after being exposed to PNIPAM-BaSO₄ marker (E) [Each data point represents the average ± SD of 3 independent replicates].

Histological evaluation of major distant organs, including the liver, kidney, spleen, and lung, revealed no observable pathological abnormalities. The liver exhibited preserved hepatic architecture with intact hepatocyte cords, normal sinusoidal spaces, and no evidence of necrosis, inflammation, or steatosis. The kidney showed normal glomerular and tubular structures, with well-defined Bowman’s capsules and no signs of tubular injury, fibrosis, or inflammatory infiltration. The lung demonstrated intact alveolar architecture with clear air spaces and thin alveolar septa, without evidence of edema, inflammation, or structural disruption. The spleen maintained normal organization of white and red pulp, with no signs of lymphoid depletion, hyperplasia, or hemorrhage. Overall, these findings indicate that the treatment did not induce detectable off-target toxicity or histopathological alterations in distant organs, demonstrating the biocompatibility of the PNIPAM-BaSO_4_ marker (Fig. 3**C**). Histological quantification of the implantation site (on a scale of 0–4), showed only minimal tissue responses (<1) in all categories. No significant differences were observed in inflammation, fibrosis/fibroplasia, necrosis/injury, edema/hemorrhage, or tissue architectural disruption between tissues adjacent to the implanted and tissue adjacent to PBS injection control. Overall, these results support the tissue compatibility of the PNIPAM-BaSO₄ formulation under the evaluated conditions (Fig. 3**D**).

### 3.6 PNIPAM-BaSO₄ marker exhibits no detectable cytotoxicity in vitro

The PNIPAM-BaSO₄ formulation demonstrated favorable biocompatibility in vitro. Cell viability remained unchanged in SW480 and COLO 205 cells across all volumes, with viability largely maintained at approximately 80–110% (Fig. 3**E**).

## 4 Discussion

One of the most clinically significant implications of this work is the possibility of re-introducing radiotherapy as a meaningful therapeutic modality for loco-regional colon cancer [29,30]. Unlike rectal cancer, where radiotherapy is routinely incorporated into standard-of-care management [12,13], radiotherapy has historically played only a limited role in colon cancer treatment [14,15]. This discrepancy is not because colon cancer cells are intrinsically radioresistant, as multiple studies have demonstrated substantial radiosensitivity of colon cancer cells [16,17], but rather because accurate targeting of tumors within the highly mobile colon remains extremely challenging. Continuous movement caused by peristalsis, bowel motility, and respiration increases uncertainty in tumor positioning and raises the risk of radiation exposure to surrounding radiosensitive tissues [19,20]. Consequently, treatment strategies for Stage II–III colon cancer have relied predominantly on systemic chemotherapy rather than loco-regional radiation approaches [5,31].

This study directly addresses this longstanding limitation by providing a stable and image-visible marker capable of enabling precise localization of mobile tumors over extended periods. We developed a thermo-responsive, CT-visible, injectable fiducial marker composed of PNIPAM loaded with BaSO₄, designed to improve the localization of highly mobile tumors such as colon cancer during image-guided radiotherapy. The PNIPAM-BaSO₄ marker demonstrated several clinically desirable characteristics simultaneously, including easy injectability at room temperature, rapid in situ solidification at physiological temperature, strong CT visibility, prolonged stability, and excellent biocompatibility both locally and systemically. Collectively, these findings support the potential of this platform as a practical translational solution for improving the precision radiotherapy of colon cancer.

This platform was intentionally designed with clinical simplicity and translational feasibility in mind. The PNIPAM-BaSO₄ marker could be pre-filled and injected through a standard fine-gauge syringe at room temperature and rapidly solidifies within approximately 15 s upon exposure to body temperature (Fig. 1). The approximately 15-seconds gelation time may provide a practical balance between sufficient working time for administration and rapid solidification after placement to limit diffusion. However, delivery through an endoscopic injection catheter was not evaluated in the present study, and warming of the formulation during administration could potentially alter its viscosity or initiate gelation before placement is completed. Future studies should therefore evaluate the working time and injection performance under clinically relevant endoscopic delivery conditions and, if necessary, optimize the formulation to adjust gelation kinetics. This ease of administration represents a major practical advantage compared with many existing localization strategies that require complex implantation procedures, specialized instrumentation, or surgically placed fiducials [26,27]. In principle, the PNIPAM-BaSO₄ formulation could potentially be delivered during a routine colonoscopy session using minimally invasive techniques already widely practiced in gastroenterology [22,23]. The intended application of the PNIPAM-BaSO₄ marker is primarily as a radiographic reference for interfractional tumor localization during treatment planning and prior to radiation delivery, rather than for intrafractional motion monitoring or real-time tumor tracking during beam delivery. Such simplicity may facilitate broad clinical adoption without major changes to existing workflows or infrastructure.

The strong CT visibility observed both in vitro and in vivo further supports the suitability of this system for image-guided radiotherapy applications (Fig. 2). The robust dose-dependent increase in Hounsfield units with increasing BaSO₄ concentration and injected volume demonstrates the radiopacity and clear visibility of the marker using micro-CT imaging, supporting its potential applicability in clinical CT imaging systems, which remains to be evaluated in future studies. This is particularly important because CT imaging remains the backbone of radiotherapy planning and treatment guidance worldwide [22,32,33].

Unlike conventional endoscopic tattooing agents such as India ink or methylene blue, which are poorly visualized radiographically and may diffuse over time [34,35], the PNIPAM-BaSO₄ marker maintained stable localization and signal intensity for months (Fig. 2). Such prolonged stability is clinically meaningful because radiotherapy regimens are typically administered over multiple treatment sessions across several weeks [24]. A stable fiducial marker minimizes the need for repeated procedures and reduces uncertainty in treatment planning.

Equally important, the PNIPAM-BaSO₄ marker demonstrated excellent biocompatibility (Fig. 3). Histopathological analysis revealed no detectable local tissue damage, inflammatory infiltration, fibrosis, or necrosis at the implantation site, while distant organs showed preserved normal architecture without evidence of systemic toxicity. These findings are particularly encouraging given the intended use of the marker in the gastrointestinal tract, where local inflammation or tissue injury could have significant clinical consequences. The absence of observable toxicity supports the feasibility of repeated or prolonged implantation if clinically required. A limitation of this study is the relatively small number of biological replicates, particularly in the in vivo experiments, and larger studies will be needed to further validate the long-term stability and biocompatibility of the fiducial marker.

Beyond colon cancer, this technology may have broader implications for the radiotherapy of other highly mobile tumors. Tumors located within the gastrointestinal tract, lung, liver, or other anatomically dynamic organs frequently present similar localization challenges during radiation treatment [22,36,37]. Therefore, a simple injectable marker capable of rapid in situ solidification and long-term radiographic visibility could potentially improve targeting accuracy across multiple disease settings. Furthermore, because the marker remains localized and structurally stable, future iterations may incorporate additional imaging functionalities or therapeutic payloads to further expand clinical utility.

An important strength of this system is its multimodal translational compatibility. BaSO₄ is already extensively used clinically as a radiographic contrast material in gastrointestinal imaging because of its excellent radiopacity and established safety profile [38,39]. Likewise, PNIPAM-based thermo-responsive biomaterials have been widely investigated for biomedical and drug delivery applications because of their predictable phase-transition behavior and favorable biocompatibility characteristics [[40], [41], [42], [43]]. The use of two well-established clinically relevant materials substantially lowers translational barriers and may accelerate future regulatory development compared with entirely novel synthetic systems. From a commercialization and clinical implementation perspective, this is highly advantageous since this technology can integrate seamlessly into existing imaging and treatment platforms.

This study has several limitations. The current work focused primarily on providing a proof-of-concept for the development of the PNIPAM-BaSO₄ marker and its characterization, including injectability, imaging visibility, stability, and biocompatibility. Future studies should evaluate performance after injection into the colon wall in larger animals and directly assess its ability to track colon movements. Future studies should also evaluate marker visibility and localization performance using clinically relevant radiotherapy imaging systems and patient-equivalent phantoms before translation to clinical IGRT applications. In addition, while subcutaneous implantation demonstrated excellent stability and safety, studies within the gastrointestinal wall and under physiologic bowel motion conditions will be important to further validate translational applicability. Long-term biodegradation behavior and elimination kinetics should also be investigated in future preclinical studies, especially in larger animals. Large animal studies are planned as part of the IND-enabling data package, which will also include deeper characterization of toxicity and stability.

In conclusion, we developed a simple, injectable, thermo-responsive, CT-visible fiducial marker with strong stability and excellent biocompatibility. By providing a stable and CT-visible marker with potential application in tumor localization, this platform may help address one of the major historical barriers limiting the use of radiotherapy in colon cancer. The simplicity of administration, compatibility with existing clinical imaging infrastructure, and use of clinically established materials such as BaSO₄ and PNIPAM collectively support the further development of this technology for potential application in image-guided radiotherapy.

## Ethics declaration

This study was conducted in accordance with the ARRIVE (Animal Research: Reporting of In Vivo Experiments) guidelines. This study was approved by the Institutional Animal Care and Use Committee (IACUC) at UT Southwestern Medical Center. (Approval No 103,868)

## CRediT authorship contribution statement

**Saeideh Arsalani:** Writing – original draft, Formal analysis, Data curation, Conceptualization, Methodology, Writing – review & editing, Visualization, Investigation. **Alireza Zomorodian:** Writing – original draft, Data curation. **Mina Maksimos:** Data curation. **Saad Asim:** Data curation. **Tithi Bhattacharyya:** Data curation. **Yun-Cheng Liu:** Data curation. **Obed Asare:** Data curation. **Feda Azab:** Data curation. **Anne M. Alsup:** Data curation. **Muhammad Rizwan:** Resources. **Emina Huang:** Writing – review & editing. **Abdel Kareem Azab:** Writing – review & editing, Writing – original draft, Visualization, Validation, Supervision, Resources, Project administration, Methodology, Investigation, Funding acquisition, Formal analysis, Conceptualization.

## Declaration of competing interest

Abdel Kareem Azab and Saeideh Arsalani filed a provisional patent describing this technology. All other authors declare no competing interests.

## Data Availability

Data will be made available on request.
